# Canine Rabies and a Case of Hydrophobia

**Published:** 1902-07

**Authors:** R. Menger

**Affiliations:** San Antonio, Texas


### TEXAS MEDICAL JOURNAL.

ESTABLISHED JULY, 1885.

PUBLISHED MONTHLY.—SUBSCRIPTION $1.00 A YEAR.

Vol. XVIII. AUSTIN, JULY, 1902. No. 1.

### Original Contributions.

For Texas Medical Journal.

### Canine Rabies and a Case of Hydrophobia.

BY R. MENGER, M. D., SAN ANTONIO, TEXAS.

During the last three months San Antonio, as well as some other cities in Texas, has been infested with an epidemic of a peculiar disease among the canine species, resembling in some case genuine rabies, and in other, and the majority of afflicted dogs, a croupous laryngeal or bronchial affection prevailed, according to the statements of local veterinarians. That, as stated, some of the canines have had genuine rabies there hardly seems to be any doubt, as some other animals bitten by such diseased dogs contracted symptoms and death from hydrophobia, and lately an aged man died from hydrophobia, after having had his left hand lacerated over two months previous by a mad dog.

San Antonio had several such "mad dog scares" during the last thirty or more years, and Dr. F. Herff, in a private letter to the writer some time ago, when relating his observations on the ecchinococcus disease in Texas, also mentioned that in 1874 and before that time San Antonio had an epidemic of canine rabies, and Dr. F. Petersen, then city physician, had furnished a number of interesting material on the matter. (Incidentally, I will state the observations of Dr. Herff in connection with the rabies scare in 1874, when Herff was also experimenting with intestinal trichinæ and the taenia ecchinococcus, at a time when also several serious cases of trichinosis occurred in San Antonio: "The taenia acchinococcus, the smallest tapeworm species known, lives in the intestine of the dog and it is very difficult to find on account of its small size and the roundish appearance of its body, resembling at first sight a small nematoid worm. I stumbled on it during the examination I made on a dog which I had fed with trichmotic sausage while hunting for intestinal trichinæ, and on first sight I took it erroneously for that animal. Afterwards I found it many times in the intestines of many dogs which were killed by the police during the rabies scare and furnished me by Dr. Petterson, then city physician, some twenty-eight years ago," etc.)

A few fatal cases of hydrophobia in human subjects, according to official records of our city, occurred at that time. Later on sporadic cases and epidemics of canine rabies happened, but none to my recollection in any way as numerous as our late epidemic, the majority of which being cases of some epizootic laryngeal disease among the dogs. Thus epizootic canine disease affects, it seems, the nerve centers of the dog and salivary glands, besides causing the croupous affection of the larynx. If, now, "rabies" is a distinct disease, independent of this epizootic, I am, for want of authoritative veterinary material, not able to state; but it certainly would have been a gain to our knowledge had post-mortems and bacteriological tests and inoculations been made, as there has been an over-abundance of experiment material at hand.

Our late epidemic aroused considerable interest, and, as usual also among the laity, as stringent measures (and the usual "kicks" of dog owners) were enacted by the city officials at the beginning of the epidemic. From the voluntary statements of local veterinarians it appears that the majority of the diseased dogs are afflicted with a croupous laryngitis and other complications. Still, in order that the public be on the safe side, I deemed it advisable to publish the following in a local paper, which was approved by my friend, health officer Dr. Paschal:

"THE LATE EPIDEMIC OF A PECULIAR DISEASE.

"The late epidemic of a peculiar disease among the canine species in our city and other places, producing a condition of madness among dogs, and even cats or other animals, just at this season of the year is quite an unusual occurrence, but, nevertheless, a fact, and it has led to various inquiries and explanations, so far, though, still veiled in some obscurity for want of expert investigation. Whether the droughty season or some peculiar atmospheric influences, want of water or moisture or other influences, are the cause is still unsettled. From the valuable reports of some of our local veterinarians, it seems the majority of the afflicted dogs show symptoms of an acute laryngitis, with secondary symptoms, such as brain and spinal congestion and irritation, with paralysis in some instances, obstruction of the air passages, etc. Still, no post-mortem examination has been made in any of the numerous fatal cases with subsequent bacteriological tests—the latter, of course, only strictly in the interest of science. Now, without wishing in any way to pose as an alarmist or to belittle the opinion of others, I would advise to not take the matter too leniently, because the dogs, the majority at least, are, or have been, afflicted with a throat disease—but to the contrary, to consider any laceration from a dog bite as very serious, and to remember that it is always better to be on the safe side and use all precautions possible. Our city officials have done the right thing in ordering dogs to be kept secluded in the yard (where they belong) or to have them muzzled, and have those killed roaming about and showing symptoms of madness—whether it be genuine hydrophobia or not. So far, no case of hydrophobia in any of the bitten subjects has occurred, and it is hoped never will occur; but statistics prove that the disease even from slight lacerations may lay dormant for a long time, months and years, before rabies develop, and as seen, it is not always at the time of accident possible to determine whether the biting dog was afflicted with genuine rabies or not."

The above was written by me about four days ago, when on April 6th a genuine case of hydrophobia and death occurred here in a German, aged 59 years. The victim was bitten by a dog on January 15th and yesterday, about eighty days after the laceration, the man died under all symptoms of hydrophobia. It seems the victim attempted to chain the diseased dog, but he resisted and bit him on the left wrist. His wife ran to his assistance, and she, also, was bitten on the hand. The dog escaped, but was later on killed. Dr. M. Bliem attended the case, also later on Dr. F. Paschal was called in consultation, and both pronounced it hydrophobia. The victim in attempting to drink a glass of beer failed to do so; he was seized with a spasmodic attack, went home later on, but was seized again with spasms and choking sensations, with froth at the mouth when water was offered him. His condition grew steadily worse until his last day, when under fearful convulsions coma set in and ended his life. Dr. Paschal, I understand, made an appeal for a post-mortem examination in order to remove a portion of the spinal medulla, but it was not granted by the relations.

P. S.—As indicated in the daily press, reports of rabies among different species of animals has been unusually prevalent in the late droughty months throughout Texas, especially among the prairie wolf, and a fatal case of hydrophobia was reported recently from a small Texas town where a farmer, an aged German, was attacked by a wolf and had died from hydrophobia several weeks later. This farmer, it was reported, had killed a wolf roaming about his farm, and hardly was this one killed when the farmer was attacked and bitten by another wolf. A short time afterward symptoms of hydrophobia developed and he died under fearful paroxysms of convulsions.

At this date of writing, May 15th, another fatal case of hydrophobia occurred in San Antonio. A small child, aged two years, in playing on the sidewalk, was bitten by a large dog in the arm and died yesterday under continual convulsions and intense suffering for the two days previous. The lascerated wounds were promptly cauterized, the wound healed, and nothing was thought of the matter any more until a few days ago when the child was suddenly attacked with convulsions and all symptoms of hydrophobia, lasting three days.

Another victim of hydrophobia from canine rabies occurred yesterday, June 16th, in the person of a bright young lady, lately graduated from our high school. According to accounts, the case was a very sad one indeed. She was bitten three weeks ago, the wounds were attended to promptly, healed, and nothing was suspected until recently, when symptoms of hydrophobia set in, from which she died under fearful convulsions. This is the third case in San Antonio since the last six weeks. The city officials have been very vigilant in killing all reported and suspected sick canines, and the epidemic is about over, and there has not been in the history of our city as many afflicted dogs killed as this year.

